# Access to cervical screening in Australian general practices: a cross-sectional study using a ‘secret shopper’ approach

**DOI:** 10.1007/s10552-026-02215-3

**Published:** 2026-07-22

**Authors:** Lucy Boyd, Claire Nightingale, Javiera Martinez-Gutierrez, Ana Machado Colling, Angela Kelly-Hanku, Paula Jops, Claire Bavor, Maleeha Ashfaq, Claire Zammit, Kristy Meiselbach, Julia Brotherton

**Affiliations:** 1 Centre for Health Policy, Melbourne School of Population and Global Health, Faculty of Medicine, Dentistry and Health Sciences, The University of Melbourne, Melbourne, Australia; 2 Department of General Practice and Primary Care, Faculty of Medicine, Dentistry and Health Sciences, The University of Melbourne, Melbourne, Australia; 3 Department of Family Medicine. Faculty of Medicine, Pontificia Universidad Católica de Chile, Santiago, Chile; 4 Kirby Institute, University of New South Wales, Sydney, Australia

**Keywords:** Cancer, Primary care, Screening, Health services research, Women’s health, Policy

## Abstract

**Purpose:**

Cervical screening in Australia is currently accessed through a healthcare provider, typically a general practitioner in primary care. We aimed to assess the real-world availability of and access to cervical screening services, including self-collection.

**Methods:**

Cross-sectional study was conducted using a secret shopper methodology with a standardized telephone script that emulated real-life calls. A random sample of Australian general practices, from each State/Territory (contacted November 2024-February 2025).

**Results:**

Of the 310 general practices, 72 (23%) could not offer a cervical screening appointment for new patients. Among practices able to provide an appointment, 217 (89%) said self-collection was available. By jurisdiction, the proportion of practices reporting availability of cervical screening ranged from 72 to 83% but did not significantly differ. The proportion of clinics that offered the choice of self-collection differed significantly by State/Territory ($$\chi_{7}^{2}$$=15·774, *P* = ·013), ranging from 60 to 100%. More regional clinics (94%) offered self-collection compared to metropolitan clinics (85%)(*P* = ·017). Practices located in the most disadvantaged socioeconomic quintile had a significantly higher proportion offering self-collection (95%) compared to those located in the most advantaged quintile (79%)(*P* = ·043). Out-of-pocket or gap fees were charged by 183 (75%) clinics (average: $AU50·73, range $AU15-$94·15).

**Conclusion:**

Our study highlighted that access to cervical screening may be constrained by limited appointments for new patients, out-of-pocket costs, and other factors. These barriers may disproportionately affect individuals facing structural and financial disadvantage. We found significant variability in availability of self-collection by location. Exploring flexible models, including mail-out, pharmacy supported, or community-led models, may improve accessibility and reduce burden on general practices.

**Supplementary Information:**

The online version contains supplementary material available at 10.1007/s10552-026-02215-3.

## Introduction

Finding an appropriate service and making an appointment is an often-overlooked component of primary healthcare access [1, 2]. In Australia, general practices (GP clinics) are the entry point for most people seeking medical care. General practitioners (GPs) are community-based doctors who provide comprehensive medical care and care coordination. Patients can attend any practice of their choosing, although many people have a preferred GP (79%) and only two in three report that they can see their preferred GP when needed [3]. GP clinic receptionists are often an individuals’ first point of contact with the service, providing essential information about how clinics work or the cost and may be the only person available to answer questions. The notion of medical receptionists as the gatekeepers to health professionals remains, even in an era of online booking systems and telehealth [4]. While this oversimplifies a broader system issue, it reflects the reality that accessing a healthcare professional can be difficult [1, 4]. These difficulties likely impact all healthcare access [2] but are especially important for preventive care and regular health checks that may not be prioritized by individuals, such as cervical screening tests (CSTs).

Australia’s National Cervical Screening Program (NCSP) offers 5-yearly CSTs for people aged 25–74 years and has successfully reduced incidence and mortality from cervical cancer [5, 6]. A CST is a primary test for oncogenic human papillomavirus (HPV) with partial genotyping, followed by liquid-based cytology triage if positive [6]. Most cervical screening occurs in GP clinics, to which there are known access issues impacting Australians in relation to distribution, availability, and patient borne costs. Medicare (Australia’s universal public health insurance scheme) supports primary care by providing a rebate or set fee toward the cost of each service. Where a practice charges above this fee, patients are left ‘out-of-pocket’ as the Medicare rebate is less than the amount charged [3].

While overall participation in the NCSP is high, with 5-yearly participation over 70% in 2024, inequities remain [7]. People living in rural and remote areas, and in areas with a lower socioeconomic (SES) index, have lower rates of participation [7]. The NCSP is also not as effective in reaching Aboriginal and Torres Strait Islander people, multicultural communities, people with disability and LGBTQ + people [7, 8].

In 2022, the choice of HPV self-collection, whereby screening participants collect their own vaginal sample rather than undergoing a speculum examination, was implemented for all eligible people within the NCSP. Self-collection mitigates many well-described barriers to screening [9], providing a more convenient, comfortable and less invasive way to screen [10]. It has increased participation among under- and never-screened individuals in Australia and internationally [11–13]. However, in Australia, there is an ongoing requirement for the CST, even when self-collected, to be ordered and overseen by a healthcare professional (currently a doctor or nurse practitioner), which could be creating additional access barriers [14].

In this novel study, we used a ‘secret shopper’ (simulated/standardized patient[15–19]) design aiming to understand real-world availability of, and access to, cervical screening (including self-collection) in Australian general practices.

## Methods

### Study design

Utilizing a cross-sectional survey design, researchers posing as prospective patients used a standardized script to call a randomly selected list of Australian general practices between November 2024 and February 2025. The script emulated real-life calls and sought specific information about access to cervical screening including self-collection (see Online Resource 2).

### Sample

The sampling frame used the Healthdirect-National Health Services Directory (NHSD) 2023 dataset [20]. We included records identified as general practices inclusive of Aboriginal medical services, sexual health clinics, and privately operated practices. Suburbs and postcodes with ≥ 1 general practice listing were included. Records were stratified by State/Territory, and the most populous states (New South Wales, Victoria, Queensland, Western Australia and South Australia) were divided into metropolitan and regional postcodes. Postcodes were divided according to the Modified Monash Model which measures remoteness and population size on a scale of Modified Monash categories 1–7. MM 1 is a major city and MM 7 is very remote. For the purposes of our study, we classified MM1 and MM2 as metropolitan and MM3 + as regional [21]. Stratification was performed in order to provide an adequate sample size for analysis from these areas and from which the random sample of practices to call was generated. Further detail on sample selection is outlined in Online Resource 1.

### Scoping and script development

 GP researchers reviewed the study protocol and provided feedback on its acceptability. A standardized script was developed and pilot tested with three clinics and a GP clinic receptionist. The script was refined until consensus was reached among authors. Emphasis on emulating a real-life call was maintained as confirmed by the receptionist.

### Data collection

Trained callers (LB, CB, CZ, MA, AMC, AH, KM, PJ, JMG) were allocated a subset of locations to call from a list of postcodes/suburbs. Calls were made on different days and times, acknowledging local time zones. Callers received a training manual and protocol and entered data into a structured REDCap® [22] database hosted securely at The University of Melbourne, which included branching and the relevant script for different scenarios. Responses were recorded via multiple choice or tick boxes and fields for free text if required. The script introduced the caller as a new patient to the clinic seeking to ask a few questions about cervical screening (see Table 1 for list of questions and details recorded). See Online Resource 2 for all questions and response options.

**Table 1:** Questions asked and details recorded during calls

Questions asked during phone call
Does the clinic offer cervical screening?
Do they offer self-collection as an option?
Can my appointment be with a female provider?
Can my appointment be with a nurse instead of a GP?
Is this a bulk billing^a^ (no patients pay out-of-pocket^b^ costs), mixed billing or private billing (all patients pay out-of-pocket^b^ costs) clinic?
What is the out-of-pocket^b^ and upfront^c^ charge for a cervical screening appointment?
What is the process of receiving results? Is there an additional cost for this?
Are appointments available outside regular business hours (9am-5pm Mon-Fri) including on weekends?

Other details collected
Date and time of call
Does the clinic have a website?
Does the website have any information about cervical screening and/or self-collection?
Does the clinic have an online booking system?

Details NOT collected
Clinic name or address
Name of any individual GPs or any other staff
Potentially identifiable information

^a^Bulk billing refers to when the GP clinic/provider accepts the Medicare rebate as the full fee for service. There are no upfront or out-of-pocket costs for the patient

^b^An out-of-pocket cost or ‘gap’ fee is paid by the patient when the practice charges a higher fee than the scheduled fee for a service. The patient pays the difference between the total fee charged and the Medicare rebate

^c^Upfront charges occur when the patient pays the total fee for service (out-of-pocket/gap fee plus Medicare rebate) and they are subsequently reimbursed the Medicare rebate

### Analysis

Practices were assigned to a SES quintile using the Socio-Economic Indexes for Areas (SEIFA) Index of Relative Socio-Economic Advantage and Disadvantage (IRSAD) index for postal area code [23]. Data were analyzed using IBM SPSS Statistics (Version 30). Analysis used descriptive statistics, with Pearson’s Chi-squared tests or Fisher’s exact tests for association to assess whether geographical characteristics were significantly associated (*P* ≤ 0·05) with cervical screening or self-collection availability. For these analyses, ambiguous responses (e.g., ‘unsure’/ ‘would need to ask the GP’) were grouped with the ‘no’ responses, to reflect the information and choice (lack of) provided to the caller.

### Ethics

As the study investigated access to health services and did not collect any identifiable information, it was considered exempt from ethics approval by The University of Melbourne Human Research Ethics Committee. The study protocol was shared and following review, a letter of exemption provided (supplementary material 3). Although exempt from ethics, the authors carefully designed this study to reduce and mitigate potential harms. The key ethical issue associated with a secret shopper methodology is deception or concealment. Steps were taken to ensure that no identifiable information was collected during the calls. For example, names and numbers of GP clinics or people answering the phone were not recorded, only the postcode and suburb they were located in. This greatly reduced the likelihood that data collected could be traced back to a specific GP clinic as many postcodes and suburbs had multiple results.

The funder of the study had no role in study design, data collection, data analysis, data interpretation, or writing of the report.

## Results

**Fig. 1:**
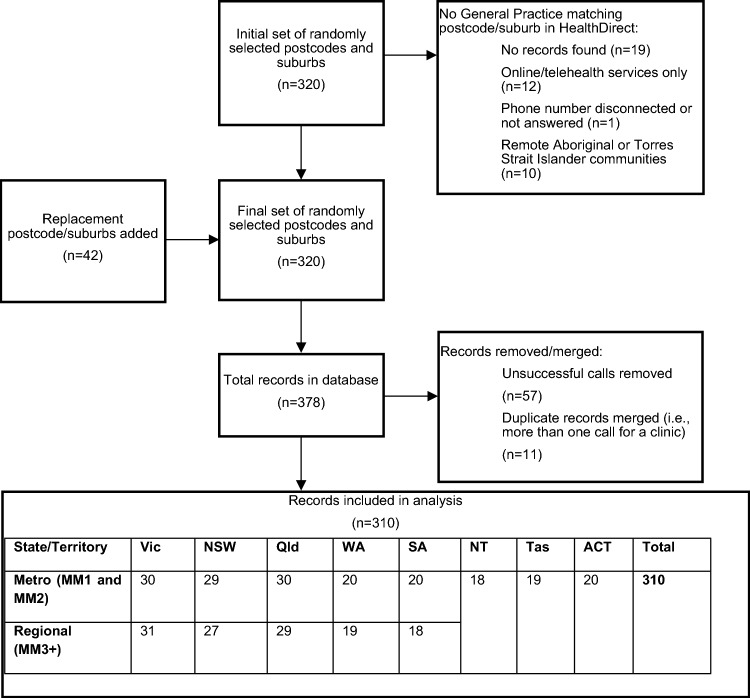
Flowchart of calls and records

There were 378 records from single call attempts made between 14th November 2024 and 24th February 2025. Records of unsuccessful calls (*n* = 57) were removed and duplicate records (*n* = 11) merged, leaving 310 successful, unique records. Duplicate records occurred when a clinic asked to call back or the call was accidentally disconnected. The 310 calls were spread across each State/Territory and region. See Fig. 1.

Of 310 clinics, 72 (23%) could not offer a cervical screening appointment for a new patient. Either they were not taking new patients (*n* = 53, 17·1%), did not offer cervical screening (*n* = 8, 2·6%) or were unsure if they offered cervical screening (*n* = 5, 1·6%). Six (1·9%) were classed as ‘other,’ including four only offering self-collection (not offering clinician collection) and having a visiting healthcare provider (HCP)/clinic available periodically (i.e., not providing a regularly accessible service). Three of the eight clinics who did not do CSTs did not provide any information as to why: for example, one respondent stated, “We just don’t do it here.” Two clinics said they provided care only for or under specific conditions (i.e., dialysis patients or occupational health/pre-employment checks) and did not provide regular general practice services. One clinic stated that the only doctor trained to provide cervical screening tests no longer worked there. One said they only provide telehealth services, although this was not communicated online. One stated they were awaiting delivery of testing kits to begin offering cervical screening again.

Most clinics offering screening offered self-collection (*n* = 217, 88·9%). Four (1·6%) stated they do not offer self-collection, 12 (4·9%) said ‘would need to discuss it with the HCP’ and 10 (4·1%) were ‘unsure.’ One clinic (0·4%) was marked as ‘did not ask/discuss.’ Of the four that did not offer self-collection as a choice, callers were given brief explanations: one staff member stated self-collection was not possible for a CST and three suggested self-collection was inferior to clinician collection (Box 1).

**Box 1:** Statements documented by callers for why self-collection is not offered

Reasons provided for why self-collection is not offered:
“if it were me, I wouldn’t do it [self-collection]”
“does not offer self-collection because Drs that work here believe they collect it better, bit of a hit and miss with self-collection and swabbing the right area(…) Less accurate whereas clinician-collection is 100%.”
“It’s not actual cervical screening at all”
“The best way to do it, is through proper Pap smear with speculum”
“it only tells you if you have a virus, nothing else”
“you are better off having the Pap smear, it will look at the inside and see if your cells are not normal”

By jurisdiction, the proportion of practices reporting availability of cervical screening ranged from 72% in WA to 83% in NT but did not significantly differ by State/Territory (*P* = ·94) and was very similar in the three most populous States (NSW, VIC and QLD)(Table 2). Screening appointments were available in 82% of clinics in non-metropolitan areas and 76% in metropolitan areas (${\chi}_{1}^{2}$=1·894, *P* = ·17). There was no significant difference in cervical screening appointment availability by quintile of relative SES advantage and disadvantage of practice location (*P* = ·62).

**Table 2:** Association between geographic characteristic (state/territory, metropolitan or regional, or SEIFA IRSAD) and cervical screening appointment availability^a^

Characteristic	Cervical screening appointment available	*P*-value	Self-collection offered as a choice	*P*-value
Overall	244 (79%)		217 (89%)	
By State/Territory^b^	(*n* = 310)		(*n* = 243)	
ACT	15 (75%)	0·939	9 (60%)	0·013*
NSW	45 (80%)		39 (87%)	
NT	15 (83%)		14 (100%)	
QLD	48 (81%)		44 (92%)	
SA	31 (82%)		31 (100%)	
TAS	14 (74%)		13 (93%)	
VIC	48 (79%)		42 (88%)	
WA	28 (72%)		25 (89%)	
By metropolitan or regional^c^	(*n* = 310)		(*n* = 243)	
Metropolitan (MM1-2)	124 (76%)	0·167	105 (85%)	0·017*
Regional (MM3 +)	120 (82%)		112 (94%)	
By SEIFA IRSAD quintile^c^	(*n* = 309)^d^		(*n* = 243)	
Most disadvantaged—1	65 (83%)	0·619	61 (95%)	0·043*
2	48 (74%)		45 (94%)	
3	36 (80%)		33 (92%)	
4	39 (75%)		34 (87%)	
Most advantaged—5	56 (81%)		44 (79%)	

^a^Collapsed to dichotomy, records with ‘no’, ‘yes but not taking new patients’, ‘unsure’ or ‘you need to ask HCP when you see them’ have been considered as not providing the service. Records with ‘yes’ and ‘other’ have been collapsed as ‘yes’

^b^Fisher–Freeman–Haltman exact test (contained cells with expected count < 5). Three cells for the test by state/territory had expected counts of less than 5, therefore this test does not meet assumptions for Pearson’s Chi-squared

^c^Pearson Chi-square test for association. All expected cell frequencies were greater than five for association by metropolitan and regional areas and SEIFA IRSAD quintiles

^d^One record is missing as this postcode did not receive a SEIFA score due to postcode non-response and low population counts in Census data *Denotes *P* < ·05

The proportion of clinics that offered the choice of self-collection differed significantly by State/Territory (${\chi}_{7}^{2}$=15·774, *P* = ·013), ranging from 60% in ACT to 100% in NT and SA. More regional clinics (94%) offered self-collection compared to metropolitan clinics (85%)(Fisher’s exact test *P* = ·017). Practices located in the most disadvantaged quintile of relative SES advantage and disadvantage had a significantly higher proportion offering self-collection (95%) compared to those located in the most advantaged quintile (79%)(*P* = ·043). The Cochran–Armitage test of trend showed a statistically significant linear trend, *P* = ·002, with lower SEIFA IRSAD areas associated with a higher proportion of clinics offering the choice of self-collection.

Nearly half of the clinics stated they had a mixed billing model (*n* = 120, 49·2%), followed by private billing (all patients pay out-of-pocket costs) (*n* = 61, 25%) and bulk billing (no patients pay out-of-pocket costs) (*n* = 50, 20·5%). Thirteen clinics (5·3%) were recorded as ‘did not ask/discuss.’

Table 3 provides an overview of costs for mixed (*n* = 120) and private billing (*n* = 61) clinics. Of the 120 mixed billing clinics, ninety-seven provided upfront costs and 102 provided out-of-pocket costs. Of those that provided costs, the mean cost for mixed billing clinics was AU$100·79 upfront and AU$48·65 out-of-pocket (range AU$15–188 upfront, AU$15–90 out-of-pocket). Of the sixty-one private billing clinics, forty-seven provided upfront costs and fifty gave their out-of-pocket costs. Of those that provided their costs, the mean cost for private billing clinics was AU$107·88 upfront and AU$54·99 out-of-pocket (range AU$30–AU$200 upfront, AU$30–94·15 out-of-pocket).

**Table 3:** Costs associated with cervical screening appointments ($AUD)^a^

Billing type^a^	Mixed billing only		Private billing only		Overall (mixed and private, not bulk billing)	
	Upfront (*n* = 97, 23 missing)	Out-of-pocket (*n* = 102, 18 missing)	Upfront (*n* = 47, 14 missing)	Out-of-pocket (*n* = 50, 11 missing)	Upfront (*n* = 144, 37 missing)	Out-of-pocket (*n* = 152, 29 missing)
Mean	$100·79	$48·65	$107·88	$54·99	$103·11	$50·73
Median	$93	$45·43	$100	$53	$95	$47·08
Std deviation	$30·48	$16·77	$30·75	$15·73	$30·65	$16·65
Minimum	$15	$15	$30	$30	$15	$15
Maximum	$188	$90	$200	$94·15	$200	$94·15

^a^Bulk billing clinics (*n* = 50) and those who did not discuss costs (*n* = 13) are excluded from this table

Notably, some clinics had the same value for upfront and out-of-pocket costs because they charged a fixed fee that was not available for rebate through Medicare. For example, one clinic that advertised themselves as being ‘bulk billing’ stated they charged an extra fee for selected services, including cervical screening which was $15. Another example was a clinic that was run once every few weeks by a traveling nurse at a flat fee. Both clinics, and other similar instances, were classified as mixed billing for the purpose of our analysis.

Some clinics also provided longer responses and/or more nuanced pricing. Many clinics with missing values above stated that their fees vary depending on which doctor you see and that the final fee is at the doctors’ discretion (*n* = 29, 12%). Some (*n* = 16, 6·6%) mentioned that the out-of-pocket fee may be higher for longer appointments, whereas others (*n* = 14, 5·7%) said it was the same regardless of appointment time. Five clinics stated that the price for clinician collection was higher than self-collection, including one clinic that offered to bulk bill self-collection.

Clinics were asked if they bulk-billed for results appointments. The most common response was that this was at the discretion of the GP or HCP (n = 110, 45·1%), followed by those that bulk bill results appointments (*n* = 64, 26·2%). Almost a fifth of clinics (*n* = 47, 19·3%) said they charge a fee for all results appointments. The remaining clinics were unsure (*n* = 8, 3·3%) or were not asked (*n* = 15, 6·1%).

Most clinics said the appointment could be with a female healthcare provider (*n* = 198, 81·1%): however, some said this was not possible (*n* = 26, 10·7%). Eight clinics (3·3%) were categorized as ‘other’—all had rotating/locum GPs and could not guarantee the gender of the HCP. A further 12 clinics were not asked/did not discuss.

Most clinics could not provide cervical screening by a nurse, stating that it was only performed by a GP (*n* = 164, 67·2%). Of the 46 clinics (18·9%) who could provide nurse screening, one stated that a nurse is the only option as they only have male GPs who are not comfortable doing CSTs. Reasons for not being able to have the appointment with the nurse included: no nurses at the clinic, lost funding for nurses to do this, Medicare changed the item numbers and now nurses cannot do it, and no longer having a trained nurse (Box 1).

Many clinics recorded as unsure or other (*n* = 15, 6·1%) stated that we would need to discuss our specific situation with the GP before seeing a nurse for cervical screening or that a nurse can chaperone a GP for clinician collection. Some stated that nurses only do CSTs when a patient is booked with a male GP who does not do CSTs.

Over half of the clinics only had appointments available during business hours (9am–5pm, Mon–Fri)(*n* = 132, 54·1%). Some offered out of business hours appointments on weekends (n = 28, 11·5%), weekdays only (*n* = 14, 5·7%) or both (*n* = 25, 10·2%). Thirty-one clinics were not asked/discussed, and 14 (5·7%) clinics were marked as ‘other.’ Reasons for ‘other’ included out-of-hours appointments reserved for emergencies-only; inconsistent availability of female GPs and/or not offering cervical screening; and appointments reserved for existing patients. One clinic said they can arrange out-of-hours at-home visits.

Most had appointments available within 1 week (*n* = 107, 43·9%) or within 48 h (*n* = 73, 29·9%). However, 26 (10·7%) stated the wait would be over 2 weeks with 12 (4·9%) recorded as did not ask/discuss.

## Discussion

Our ‘secret shopper’ study provided insights into cervical screening access in Australian general practice that may not have been possible to capture via other methods. Studies using a similar methodology have been conducted in other settings, [2, 16–19] but to the best of our knowledge, this is the first study using this method to examine access barriers to cervical screening. We found that 1 in 5 practices were unable to offer a caller a cervical screening appointment. While appointment access was similar by State/Territory, by region, and SES area, there were significant disparities in the availability of self-collection. Self-collection availability ranged from 60% in the ACT to 100% in NT and SA and was higher in regional compared to metropolitan areas and in more disadvantaged than more advantaged SES areas.

National data on the proportion of CSTs that were clinician-collected compared to self-collected show similar patterns. In the same quarters in which this study was conducted (Q4 of 2024 and Q1 of 2025), the proportion of tests that were self-collected nationally was 40·4% and 42·7%, respectively [24]. Most recent data indicate 46% of tests are now self-collected and, although the overall number of tests has decreased, the trend of increasing proportion of self-collected tests continues [24]. The proportion of self-collected tests varies by State, rurality, and SES with the most pronounced differences occurring by State and Territory. In Q4 2024 and Q1 2025, TAS had the highest proportion of self-collected tests (53·36% and 55·02%), followed by NT and VIC, and the ACT had the lowest proportion of self-collected tests (32·14% and 35·70%) [24]. Our findings indicate that variations across State/Territory, regionality and SES are likely influenced by differences in access to self-collection, rather than solely by participants’ preferences. Several comments made about self-collection also suggest that some GPs still have concerns about the accuracy and role of self-collection, as documented previously, highlighting the known challenges in attempting to change long-standing routine clinical practices [25]. These findings signal a need to consider additional strategies, such as provider education and laboratory support, to improve the availability of self-collection in some areas, although it is notable, and somewhat reassuring, that in areas with historically lower screening participation, self-collection availability appears to be higher.

Other potential barriers were identified, including wait time until an appointment, cost of appointment, and lack of choice of provider. While wait times for an appointment were mostly under two weeks, some clinics had longer periods, possibly indicative of wider system issues. Most stated they only had appointments available during business hours which has been documented as an additional barrier particularly for those who have work or carer responsibilities [26–28]. This is another indicator that flexible or community-led models are required, instead of expecting general practice to operate out of hours, the system could reduce pressure by shifting demand to services that are already open/accessible at other hours. A successful example of task shifting from general practice to community pharmacy is in urinary tract infection (UTI) diagnosis and treatment. Recently, several states have commenced a community pharmacy model of prescribing antibiotics for uncomplicated UTIs and found this to be safe and effective [29–31]. Despite favorable community attitudes to cervical screening kit collection from pharmacies, they are not currently available through Australian pharmacies [32]. However, many community organizations serving specific populations, (e.g., Aboriginal and Torres Strait Islander people, migrants, gender and sexuality diverse people) are already providing screening in locations beyond traditional clinics such as at community events and pop-up cervical screening clinics [33, 34].

The average upfront cost for a cervical screening appointment in our sample was AU$103·11, which is likely to be unaffordable for many screen-eligible people given screening is not an acute health care need. In a recent report, 7·7% of people said they have delayed accessing GP care due to cost. The proportion of people delaying care due to cost was highest in the 25–34-year-old group (13%) [3]. We also noted complexity and lack of clarity in pricing schedules for some clinics. We hypothesize that this uncertainty may be an additional, underexplored barrier for some screening participants, particularly those experiencing financial distress, in lower SES areas or in some age groups. Further research is required to understand the impact of cost, and transparency in reporting of pricing, on accessing cervical screening as well as whether cost may be a barrier to accessing follow-up care when required, given 20% of clinics charged a fee and many had no set approach.

Our study showed that some clinics could not offer appointments with a female provider and/or a nurse instead of a GP. The reasons clinics provided during our phone calls for not having a nurse appointment for cervical screening align with barriers highlighted in previous studies. These include training, a recent change to the funding model in Victoria [35], and minimal financial incentives which may all be barriers to leveraging nurses in screening [36, 37]. Under the current funding model, providers receive payment for cervical screening appointments through the MBS. GPs, other similarly qualified doctors, and nurse practitioners can access a fee for their consultation, but nurses or other providers cannot. This lack of access to appropriate remuneration, despite many cervical screening trained nurses across the country, is amplified in nurse-run health services, or clinics without a regular or permanent GP [36].

Overall, new models of remuneration for the range of health care providers who can provide cervical screening and more flexible methods to access cervical screening, as called for in Australia’s cervical cancer elimination strategy, may be an important solution to the access barriers we identified [8].

Generalizability/representativeness of our study is limited by the sample size: 310 of approximately ~ 7100 general practices in Australia. However, our methodology and randomized selection of practices reduced selection bias compared to other methods which require voluntary participation. Because some clinics were not asked all the questions and some calls were terminated quickly by the clinic due to the receptionists’ competing demands or lack of time, some variables were impacted by missing data. The authors believe this to be indicative of a system failing, which would have impacted a real consumer’s ability to make an appointment appropriate for their needs, rather than a flaw in methods.

## Conclusions

Our study highlights numerous factors impacting access to cervical screening. The high overall penetration of self-collection, and higher uptake in less accessible and more disadvantaged areas are positive reflections of the policy change. However, almost 20% of clinics were not taking new patients or did not provide cervical screening with many charging substantial out-of-pocket fees and unable to provide out-of-hours appointments. Exploring flexible ways to access cervical screening including mail-out, pharmacy supported, or community-led models is warranted to improve access and reduce burden on general practices.

Since the inception of the NCSP in 1991, cervical screening has been firmly embedded in general practice in Australia. While this has long been a strength, increasing access barriers and the availability of equally effective methods of screening that do not require a clinical examination, demand we start to provide other options to ensure screening can be accessed by all.

## Supplementary Information

Below is the link to the electronic supplementary material.

Supplementary file1 (PDF 67.0 KB)

Supplementary file 2 (PDF 173 KB)

Supplementary file 3 (PDF 130 KB)

## Data Availability

The de-identified data we analysed are not publicly available, but requests to the corresponding author for the data will be considered on a case-by-case basis. All authors had full access to the data, including statistical reports, and tables relating to the study.
