# Supplementary material for: Access to cervical screening in Australian general practices: a cross-sectional study using a ‘secret shopper’ approach
Source: Cancer Causes Control. 2026 Jul 22;37(8):129. doi: 10.1007/s10552-026-02215-3 (PMC13391652; doi:10.1007/s10552-026-02215-3)
Supplement: Supplementary file 1 — Supplementary file1 (PDF 67.0 KB) [file 10552_2026_2215_MOESM1_ESM.pdf]

Manuscript Title:

Access to cervical screening in Australian general practices: a cross-sectional study using a ‘secret shopper’ approach

Study population

A minimum overall sample size of 264 was calculated to achieve a 90% confidence level with a margin of error of ±5% around the estimated value.

Calculations to reach a minimum sample size within each state/territory were attempted however, this increased the number of calls required and proved prohibitive with available resources.

Therefore, a decision was made to have a sample of ~320 general practices (~20% greater than minimum sample size) from all Australian states and territories.

Breakdown of number of practices by state/territory and MMM:

<table>
<tr><th>State/Territory</th><th>Vic</th><th>NSW</th><th>Qld</th><th>WA</th><th>SA</th><th>NT</th><th>Tas</th><th>ACT</th><th>Total</th></tr>
<tr><td>Metro (MM1 and MM2)</td><td>30</td><td>30</td><td>30</td><td>20</td><td>20</td><td rowspan="2">20</td><td rowspan="2">20</td><td rowspan="2">20</td><td rowspan="2">320</td></tr>
<tr><td>Regional (MM3+)</td><td>30</td><td>30</td><td>30</td><td>20</td><td>20</td></tr>
</table>

Sampling frame

The sampling frame used the Healthdirect - National Health Services Directory (NHSD) 2023 dataset from the Australian Urban Research Infrastructure Network.[19] We included records identified as general practices (SNO-MED CT ID 700232004) inclusive of Aboriginal medical services, sexual health clinics and privately operated practices. Suburbs and postcodes with ≥1 general practice listing were included.

Records were stratified by state/territory, and the most populous states (New South Wales, Victoria, Queensland, Western Australia and South Australia) were divided into metropolitan (Monash Model 1 and 2) and regional postcodes (Monash Model 3-7). To select clinics, postcodes were randomised in Excel and the first 20-30 records of each list were included in the initial call list. For each location, a randomly selected ‘sequence’ number from 1-5 was chosen to identify which Healthdirect clinic would be called. For example, for sequence 3 the caller would

contact the third result that matched the postcode/suburb. If the number of results was less than the sequence number, the caller would return to the top of the list and continue counting. If only one result was present, the caller would contact this clinic. If no clinics were identified in the postcode and suburb, the caller would mark the location as 'replace' and another location from the same state/territory and rurality would be added. We aimed to include 320 practices: 30 metropolitan and 30 regional from NSW, Vic, Qld (most populous states); 20 metropolitan and 20 regional from WA and SA; and 20 metropolitan and regional combined from NT, Tas, ACT (least populous states).
