# Supplementary material for: Access to cervical screening in Australian general practices: a cross-sectional study using a ‘secret shopper’ approach
Source: Cancer Causes Control. 2026 Jul 22;37(8):129. doi: 10.1007/s10552-026-02215-3 (PMC13391652; doi:10.1007/s10552-026-02215-3)
Supplement: Supplementary file 2 — (PDF 173 KB) [file 10552_2026_2215_MOESM2_ESM.pdf]

Manuscript Title:

Access to cervical screening in Australian general practices: a cross-sectional study using a ‘secret shopper’ approach

REAL-CERV

Data Dictionary

January 12, 2026

1 Data collection

1.1 Record ID

Description:

Field Name: `record_id`

Purpose:

Data Collection: Always Collected

Data Obligation: Mandatory

Permitted Values: Text format

Data Source, Standard/ Terminology:

1.2 Team member calling:

Description:

Field Name: `caller`

Purpose:

Data Collection: Always Collected

Data Obligation: Mandatory

Permitted Values:

Code	Description
0	Lucy
1	Ana
2	Maleeha
3	Kristy
4	Claire B
5	Claire Z
6	Javi
7	Paula
8	Amelia

Data Source, Standard/ Terminology:

1.3 Date/time of call:

Description:

Field Name: `date`

Purpose:

Data Collection: Always Collected

Data Obligation: Mandatory

Permitted Values: Date and Time (DD/MM/YYYY HH:MM)

Data Source, Standard/ Terminology:

1.4 Postal code:

Description:

Field Name: `postcode`

Purpose:

Data Collection: Always Collected

Data Obligation: Mandatory

Permitted Values: Australian Postal Code

Data Source, Standard/ Terminology:

1.5 Suburb:

Description:

Field Name: `suburb`

Purpose:

Data Collection: Always Collected

Data Obligation: Mandatory

Permitted Values: Text format

Data Source, Standard/ Terminology:

1.6 Does the service have a website and/or online booking system?

Description:

Field Name: `website`

Purpose:

Data Collection: Always Collected

Data Obligation: Optional

Permitted Values:*
* multiple select

Code	Description
0	No website or online booking system found
1	Yes, website
2	Yes, an online booking system
3	Other (provide detail)

Data Source, Standard/ Terminology:

1.7 Provide detail about the website

Description:

Field Name: `website_other`

Purpose:

Data Collection: Conditional Collection

Collected When: `[website(3)] = '1'`

Data Obligation: Optional

Permitted Values: Text format

Data Source, Standard/ Terminology:

1.8 Does the website mention the availability of cervical screening?

Description:

Field Name: web_cerv

Purpose:

Data Collection: Conditional Collection

Collected When: [website(1)] = '1' or [website(3)] = '1'

Data Obligation: Optional

Permitted Values:

Code	Description
0	No mention of cervical screening, Pap test or self-collection on the website
1	Yes, website states cervical screening/Pap testing is available at this service, does not mention if self-collection is available
2	Yes, website states cervical screening/Pap testing is available at this service AND mentions self-collection is available

Data Source, Standard/ Terminology:

1.9 Provide more information if needed:

Description:

Field Name: web_notes

Purpose:

Data Collection: Always Collected

Data Obligation: Optional

Permitted Values: Notes format

Data Source, Standard/ Terminology:

1.10 Successful call?

Description:

Field Name: success

Purpose:

Data Collection: Always Collected

Data Obligation: Optional

Permitted Values:

Code	Description
0	Yes
1	No

Data Source, Standard/ Terminology:

1.11 Why?

Description:

Field Name: success_why

Purpose:

Data Collection: Conditional Collection

Collected When: [success] = '1'

Data Obligation: Mandatory

Permitted Values: Text format

Collection Guide: NOTE: THIS IS IN THE PUBLIC REPORT. NO SENSITIVE INFORMATION IN THIS FIELD

Data Source, Standard/ Terminology:

1.12 Hi there, I'mI am a new patient, (I've had a look at your website but) I had a few questions about your services. Do you do cervical screenings?

Description:

Field Name: cst_avail

Purpose:

Data Collection: Conditional Collection

Collected When: [success] = '0'

Data Obligation: Mandatory

Permitted Values:

Code	Description
0	Yes
1	No
2	Unsure
3	Will need to chat with HCP
4	Other
5	Yes, but we are not taking on any new patients

Data Source, Standard/ Terminology:

1.13 Do you provide cervical screening - other, please describe:

Description:

Field Name: cstavail_oth

Purpose:

Data Collection: Conditional Collection

Collected When: [cst_avail] = '4'

Data Obligation: Mandatory

Permitted Values: Text format

Data Source, Standard/ Terminology:

1.14 Great! Do you offer self-collection? If they ask what you mean by self-collection: I saw something on Instagram/the news/my friend told me that you can do the test yourself now, is that right?

Description:

Field Name: sc_avail

Purpose:

Data Collection: Conditional Collection

Collected When: [cst_avail] = '0' or [cst_avail] = '3' or [cst_avail] = '4'

Data Obligation: Mandatory

Permitted Values:

Code	Description
0	Yes
1	No
2	Unsure
3	Will need to chat with HCP
4	Did not ask/discuss

Data Source, Standard/ Terminology:

1.15 Do people usually do them in the practice or can I do it at home and drop it back?

Description:

Field Name: sc_homemult

Purpose:

Data Collection: Conditional Collection

Collected When: [sc_avail] = '0'

Data Obligation: Optional

Permitted Values:

Code	Description
0	In practice/clinic only
1	Can take home or do in practice
2	Other (provide detail)

Data Source, Standard/ Terminology:

1.16 Home vs in clinic notes:

Description:

Field Name: home_oth

Purpose:

Data Collection: Conditional Collection

Collected When: [sc_homemult] = '2'

Data Obligation: Optional

Permitted Values: Text format

Data Source, Standard/ Terminology:

1.17 Self-collect availability notes

Description:

Field Name: sc_notes_2

Purpose:

Data Collection: Conditional Collection

Collected When: [cst_avail] = '0' or [cst_avail] = '3' or [cst_avail] = '4'

Data Obligation: Mandatory

Permitted Values:*
* multiple select

Code	Description
0	N/A
1	Only some providers offer SC (provide detail)
2	SC only available on some days
3	will need to order in SC supplies
4	Will need to discuss with the GP
5	Other (provide detail)

Data Source, Standard/ Terminology:

1.18 Provide further details:

Description:

Field Name: sc_oth

Purpose:

Data Collection: Conditional Collection

Collected When: [sc_notes_2(1)] = '1' or [sc_notes_2(5)] = '1'

Data Obligation: Optional

Permitted Values: Text format

Data Source, Standard/ Terminology:

1.19 Do you know how long approximately those appointments take?

Description:

Field Name: appt_length_2

Purpose:

Data Collection: Conditional Collection

Collected When: [cst_avail] = '0' or [cst_avail] = '3' or [cst_avail] = '4'

Data Obligation: Mandatory

Permitted Values:

Code	Description
0	Unsure
1	6-20 mins (standard consult, item 23, Level B)
2	20+ mins (item 36, Level C)
3	40+ mins (item 44, Level D)
5	60+ mins (item 47, Level E)
4	Did not ask/discuss

Data Source, Standard/ Terminology:

1.20 And how much will that appointment cost? Do you bulk bill at all?

Description:

Field Name: cost

Purpose:

Data Collection: Conditional Collection

Collected When: [cst_avail] = '0' or [cst_avail] = '3' or [cst_avail] = '4'

Data Obligation: Mandatory

Permitted Values:

Code	Description
0	Mixed billing
1	Bulk-billing
2	Private billing only
3	Did not ask/discuss

Data Source, Standard/ Terminology:

1.21 Upfront cost ($): Approx. $ to be paid upfront, i.e., before Medicare rebate

Description:

Field Name: upfront

Purpose:

Data Collection: Conditional Collection

Collected When: [cst_avail] = '0' or [cst_avail] = '3' or [cst_avail] = '4'

Data Obligation: Optional

Permitted Values: Number

Collection Guide: Whole numbers only here, leave blank if you are given a range. Describe range in notes

Data Source, Standard/ Terminology:

1.22 Out-of-pocket cost ($): Amount patient will pay after receiving Medicare rebate

Description:

Field Name: `oop`

Purpose:

Data Collection: Conditional Collection

Collected When: `[cst_avail] = '0' or [cst_avail] = '3' or [cst_avail] = '4'`

Data Obligation: Optional

Permitted Values: Number

Collection Guide: Whole numbers only here, leave blank if you are given a range. Describe range in notes

Data Source, Standard/ Terminology:

1.23 Notes on price of appointment, mixed and bulk-billing (optional) Include here if the cost is at the discretion of the GP

Description:

Field Name: `cost_notes`

Purpose:

Data Collection: Conditional Collection

Collected When: `[cst_avail] = '0' or [cst_avail] = '3' or [cst_avail] = '4'`

Data Obligation: Optional

Permitted Values: Text format

Data Source, Standard/ Terminology:

1.24 How do you manage me getting the results? Do I have to pay for another appointment if I have to come back? Use this field for information about paying for another appointment if they come back. All other details in notes box below

**Description:**	
**Field Name:**	results_2
**Purpose:**	
**Data Collection:**	Conditional Collection
**Collected When:**	[cst_avail] = '0' or [cst_avail] = '3' or [cst_avail] = '4'
**Data Obligation:**	Mandatory

Permitted Values:

Code	Description
0	Yes
1	No
2	Unsure
3	Will need to chat with HCP
5	At the discretion of the GP/HCP
4	Did not ask/discuss

Data Source, Standard/ Terminology:

1.25 Include any other details/notes about results here (optional):

**Description:**	
**Field Name:**	result_notes
**Purpose:**	
**Data Collection:**	Conditional Collection
**Collected When:**	[cst_avail] = '0' or [cst_avail] = '3' or [cst_avail] = '4'
**Data Obligation:**	Optional

Permitted Values: Notes format

Data Source, Standard/ Terminology:

1.26 Can I have an appointment with a female HCP?

Description:

Field Name: hcp_sex

Purpose:

Data Collection: Conditional Collection

Collected When: [cst_avail] = '0' or [cst_avail] = '3' or [cst_avail] = '4'

Data Obligation: Mandatory

Permitted Values:

Code	Description
0	Yes
1	No
2	Other
3	Did not ask/discuss

Data Source, Standard/ Terminology:

1.27 Other, please explain: e.g., female is only available for full fee paying patients/female is only available on a waitlist/female only available for existing patients

Description:

Field Name: hcp_other

Purpose:

Data Collection: Conditional Collection

Collected When: [hcp_sex] = '2'

Data Obligation: Mandatory

Permitted Values: Notes format

Data Source, Standard/ Terminology:

1.28 Can I have my appointment with a nurse instead of a GP?

Description:

Field Name: nurse_2

Purpose:

Data Collection: Conditional Collection

Collected When: [cst_avail] = '0' or [cst_avail] = '3' or [cst_avail] = '4'

Data Obligation: Mandatory

Permitted Values:

Code	Description
0	Yes
1	No
2	Depends/other (describe below)
3	DId not ask/discuss

Data Source, Standard/ Terminology:

1.29 Nurse instead of GP, describe more here:

Description:

Field Name: nurse_oth

Purpose:

Data Collection: Conditional Collection

Collected When: [nurse_2] = '1' or [nurse_2] = '2'

Data Obligation: Optional

Permitted Values: Text format

Data Source, Standard/ Terminology:

1.30 How long would I have to wait for an appointment? Could I come in this week? The next available appt is:

Description:

Field Name: appt_flex_2

Purpose:

Data Collection: Conditional Collection

Collected When: [cst_avail] = '0' or [cst_avail] = '3' or [cst_avail] = '4'

Data Obligation: Mandatory

Permitted Values:

Code	Description
0	Within 48 hours
1	Within 1 week
2	In 1-2 weeks
3	More than 2 weeks
4	Did not ask/discuss

Data Source, Standard/ Terminology:

1.31 Do you have after hours appointments available?

Description:

Field Name: appt_flex_3

Purpose:

Data Collection: Conditional Collection

Collected When: [cst_avail] = '0' or [cst_avail] = '3' or [cst_avail] = '4'

Data Obligation: Optional

Permitted Values:

Code	Description
0	No, only 9-5 Mon-fri (or similar)

1	Yes, we do after hours on week days
2	Yes, we do weekends
3	Yes, we do after hours AND weekends
4	Other (describe)
5	Did not ask/discuss

Data Source, Standard/ Terminology:

1.32 Provide more info about after hours appointments:

Description:

Field Name: `appt_flex_notes`

Purpose:

Data Collection: Conditional Collection

Collected When: `[appt_flex_3] = '4'`

Data Obligation: Optional

Permitted Values: Text format

Data Source, Standard/ Terminology:

1.33 Estimated length of call (minutes)

Description:

Field Name: `mins`

Purpose:

Data Collection: Conditional Collection

Collected When: `[cst_avail] = '0' or [cst_avail] = '3' or [cst_avail] = '4'`

Data Obligation: Mandatory

Permitted Values: Number between 1 and 100

Collection Guide: round to the nearest whole number. Only include the number. For example, if your

call was 3 mins and 15 seconds you would enter "3"

Data Source, Standard/ Terminology:

1.34 Notes:

Description:

Field Name: `notes`

Purpose:

Data Collection: Always Collected

Data Obligation: Optional

Permitted Values: Notes format

Data Source, Standard/ Terminology:
