# Supplementary material for: Access to cervical screening in Australian general practices: a cross-sectional study using a ‘secret shopper’ approach
Source: Cancer Causes Control. 2026 Jul 22;37(8):129. doi: 10.1007/s10552-026-02215-3 (PMC13391652; doi:10.1007/s10552-026-02215-3)
Supplement: Supplementary file 3 — (PDF 130 KB) [file 10552_2026_2215_MOESM3_ESM.pdf]

## Lucy Boyd

**From:** Helena Clements
**Sent:** Monday, 20 May 2024 2:38 PM
**To:** Lucy Boyd
**Cc:** Erin Dyson
**Subject:** RCH0198535: Enquiry re. human research ethics review

Dear Ms Boyd

Re.: REAL-CERV: Real-world Evaluation of Access to Cervical Screening in Primary Care

Thank you for submitting this project for assessment of whether human ethics review is required. This project as described in the protocol attached has been assessed by our Office in accordance with the University's Research Ethics and Biorisk Management Policy (MPF1341). Although it does constitute research, the data that you will collect and analyse will come from and be about operational elements of the clinics themselves rather than individual people or communities. It therefore does not meet the criteria for needing human research ethics review.

Please retain this letter as a formal record of this assessment (reference number RCH0198535).

Yours sincerely

Helena Clements

**Helena Clements | Manager, Human Research Ethics | (she/her)**
Office of Research Ethics and Integrity | Research, Innovation and Commercialisation
Level 5, Alan Gilbert Building, 161 Barry Street
The University of Melbourne, Victoria 3010, Australia
**T:** +61 3 8344 3454 **E:** helena.clements@unimelb.edu.au
unimelb.edu.au

THE UNIVERSITY OF MELBOURNE

**The University of Melbourne (Australian University) PRV12150 / CRICOS 00116K**

*We acknowledge Aboriginal and Torres Strait Islander people as the Traditional Owners of the unceded lands on which we work, learn and live. We pay respect to Elders past, present and future, and acknowledge the importance of Indigenous knowledge in the Academy.*

This email and any attachments may contain personal information or information that is otherwise confidential or the subject of copyright. Any use, disclosure or copying of any part of it is prohibited. The University does not warrant that this email or any attachments are free from viruses or defects. Please check any attachments for viruses and defects before opening them. If this email is received in error, please delete it and notify us by return email.

REAL-CERV Protocol.docx
